# Supplementary material for: Positive attitudes towards feline obesity are strongly associated with ownership of obese cats
Source: PLoS One. 2020 Jun 25;15(6):e0234190. doi: 10.1371/journal.pone.0234190 (PMC7316328; doi:10.1371/journal.pone.0234190)
Supplement: S1 File — (PDF) [file pone.0234190.s001.pdf]

Australian Cat Body Composition Survey

Participant Information Statement

(1) What is this study about?
This study aims to investigate the factors that may influence the body composition of cats and cat owners' attitudes to different body compositions of cats.

(2) Who is running this study?
This study is being conducted by Kendy Teng DVM, and is part of the degree of Doctor of Philosophy in Veterinary Science at The University of Sydney under the supervision of Navneet Dhand, Associate Professor in Veterinary Biostatistics and Epidemiology.

(3) What does this study involve?
This study involves a questionnaire. Data from the questionnaire will then be analysed and reported in the form of part of Doctor of Philosophy thesis and probably journal article as well.

(4) How will I benefit from participating in the study?
Participants who complete the survey and who leave contact information will be eligible to participate in a draw to win one of the ten $50 supermarket gift vouchers (please do not repeat the questionnaire).
The research summary will be made available to participants who provide their contact details.

(5) Eligibility for Participation
Participants are required to 1) be over 18, 2) be fluent in English and 3) own at least one cat.

(6) How long will the questionnaire take?
The questionnaire should take approximately 10 minutes to complete.

(7) Can I withdraw from the study?
Participating in this study is completely voluntary and you are not under any obligation to consent to complete the questionnaire. Submitting a completed questionnaire is an indication of your consent to participate in the study. You can withdraw any time prior to submitting your completed questionnaire. Once you have submitted your questionnaire anonymously, your responses cannot be withdrawn.

(8) Is my personal information secure?
All aspects of the study, including results, will be strictly confidential and only the researchers listed above will have access to the information on participants. Individual participants will not be identifiable through the questionnaire.

(9) Can I tell other people about the study?
Yes. You are very welcome to share the questionnaire with others. Thank you.

(10) What if I require further information?
If you would like to acquire more information at any stage, please feel free to contact Dr Kendy Teng on +61 2 9351 7094 or email kendy.teng@sydney.edu.au, or Dr Navneet Dhand on +61 2 9351 1669 or email navneet.dhand@sydney.edu.au

(11) What if I have a complaint or concerns?
Any person with concerns or complaints about the conduct of a research study can contact The Manager, Human Ethics Administration, University of Sydney on +61 2 8627 8176 (Telephone); +61 2 8627 8177 (Facsimile) or ro.humanethics@sydney.edu.au (Email).

Australian Cat Body Composition Survey

* 1. How old are you?
Please be advised that you are not eligible to participate in this survey if you are under 18.

- ○ Under 18
- ○ 18-24
- ○ 25-34
- ○ 35-45
- ○ 45-54
- ○ 55-64
- ○ 65 and older

Australian Cat Body Composition Survey

Cat Number

* 2. How many cats do you own?

- ○ 0
- ○ 1
- ○ 2
- ○ 3
- ○ 4
- ○ 5
- ○ Other. How many cats?

[ ]

Australian Cat Body Composition Survey

Questionnaire Instruction

1) Please answer all the questions for 1 cat. If you own more than 1 cat - answer for the cat whose name is first when names are listed in alphabetical order.

2) We are aiming for 1 cat per household. People from the same household please avoid repeating the questionnaire.

3) The questionnaire takes about 10 minutes to finish.

Australian Cat Body Composition Survey

Demographics of Your Cat

3. Is your cat purebred or crossbred (mixed-breed)?

- ○ Purebred
- ○ Crossbred (mixed-breed)

Australian Cat Body Composition Survey

Demographics of Your Cat

* 4. What is your cat’s breed?

Other (please specify)

5. What is the hair length of your cat (or more likely to be)?

- Short hair
- Medium hair
- Long hair

6. About how old is your cat (years old)?

- ◯ < 6 months old
- ◯ 6 months- < 1 year old
- ◯ 1
- ◯ 2
- ◯ 3
- ◯ 4
- ◯ 5
- ◯ 6
- ◯ 7
- ◯ 8
- ◯ 9
- ◯ 10
- ◯ 11
- ◯ 12
- ◯ 13
- ◯ 14
- ◯ 15
- ◯ 16 and over (please specify the age)

____

7. What is your cat's sex?

- ◯ Male
- ◯ Female
- ◯ I am not sure

8. Is your cat desexed (neutered)?

- ◯ Yes
- ◯ No
- ◯ I am not sure

9. At what age was your cat desexed (neutered)?

- ◯ 0- <3 months old
- ◯ 3- <6 months old
- ◯ 6- <12 months old
- ◯ 1- <3 years old
- ◯ ≥ 3 years old
- ◯ I am not sure

10. From where did you get your cat?

- ◯ A breeder
- ◯ A pet shop
- ◯ A shelter/ pond/ rescue organisation
- ◯ A friend/ relative
- ◯ Another cat of mine gave birth to this cat
- ◯ Street
- ◯ Other (please specify)

11. Is your cat registered with Australian Cat Federation (ACF)?

- ◯ Yes
- ◯ No

12. Is your cat registered with your local council?

- ◯ Yes
- ◯ No

13. Do you have pet insurance for your cat?

- ◯ Yes
- ◯ No

14. When was the last time your cat received flea treatment?

- Less than 1 month ago
- 1- <3 months ago
- 3- <6 months ago
- 6- <12 months ago
- 1- <3 years ago
- ≥ 3 year
- My cat has never had flea treatment since I had him/her

Australian Cat Body Composition Survey

Body Composition of Your Cat

15. What's the approximate weight of your cat (kg)?

[ ]

* 16. How do you think about your cat's body condition?

- Very underweight
- Somehow underweight
- Ideal
- Chubby/ overweight
- Fat/ obese

* 17. Has any vet ever described your cat as any of the following in the past 1 year? (select all that apply)

- [ ] Very underweight
- [ ] Somehow underweight
- [ ] Ideal weight/ fit
- [ ] Chubby/ overweight
- [ ] Fat/ obese
- [ ] No vet has commented about the shape of my cat in the past 1 year
- [ ] My cat hasn't been to a vet in the past 1 year

Other (please specify)

18. How do you monitor your cat's weight?

- ○ I do not monitor it
- ○ I know the weight when I take the cat to a vet
- ○ I weigh the cat regularly
- ○ I weigh the cat from time to time, as I remember it

Australian Cat Body Composition Survey

Feeding Behaviour of Your Cat

19. How often do you feed your cat?

- ○ Once a day
- ○ Twice a day
- ○ 3 times a day
- ○ 4 times a day or more
- ○ Ad libitum

20. How often are there leftovers?

- ○ Never
- ○ Sometimes
- ○ Often
- ○ Always

21. There are few types of food here. Please rate the frequency of the type of food you feed your cat.

	Never	The minor part of the diet	The major part of the diet	The only food in the diet
Dry food (e.g. biscuits)	○	○	○	○
Canned food	○	○	○	○
Wet food apart from cans (e.g. foil packs, pouches)	○	○	○	○
Home-made cat food	○	○	○	○
Leftovers of human food	○	○	○	○
Treats/ snacks	○	○	○	○

22. How do you decide the quantity of the food? (select all that apply)

- ☐ No specific rules
- ☐ Advice from vets
- ☐ Instructions from the package
- ☐ According to the amount that the cat eats
- ☐ Feed more than the cat needs
- ☐ According to the weight of the cat
- ☐ According to the body condition of the cat
- ☐ Recommendation from Internet

Other (please specify)

____

23. Does your cat get food from neighbours and/or other outside resource?

- Never
- Often
- All the time
- I am not sure but probably yes
- I am not sure but probably not

24. Does your cat beg for food?

- Never
- Sometimes
- Often
- Always

25. How often do you give in and feed your cat when she/he begs?

- Never
- Sometimes
- Often
- Always

Australian Cat Body Composition Survey

Other Questions Related to Body Composition

26. How often does your cat spend time in each of these places?

	Never	Sometimes	Often	Always	I am not sure
Indoors					
Outdoors but in your property					
Outdoors - outside your property					

27. How often does your cat hunt?

- ◯ Never
- ◯ Rarely (Less than once a month)
- ◯ Sometimes (at least once a month)
- ◯ Often (at least once a week)
- ◯ Always (as least once every 2-3 days)
- ◯ I am not sure

28. What animals does your cat usually hunt back home? (select all that apply)

- ☐ Birds
- ☐ Larger mammals (e.g. rabbits, squirrels, possums)
- ☐ Small mammals (e.g. mice, shrews)
- ☐ Insects
- ☐ N/A
- ☐ Other (please specify)

29. How often does your cat go out during the night?

- ◯ Never
- ◯ Rarely (Less than once per month)
- ◯ Occasionally (at least once a month)
- ◯ Quite often (at least once a week)
- ◯ Always (at least once every 2-3 days)
- ◯ I am not sure

30. Please rate the extent of agreement to each statement.

	Strongly disagree	Disagree	Neutral	Agree	Strongly agree
Chubby cats are cute	◯	◯	◯	◯	◯
Fat cats are cute	◯	◯	◯	◯	◯
Chubby cats usually look happier	◯	◯	◯	◯	◯
Fat cats usually look happier	◯	◯	◯	◯	◯
Being chubby is a disease	◯	◯	◯	◯	◯
Being fat is a disease	◯	◯	◯	◯	◯
I think that it's fine for cats to be chubby	◯	◯	◯	◯	◯
I think that it's fine for cats to be fat	◯	◯	◯	◯	◯
If I have to choice, I would rather make my cat happier than fitter	◯	◯	◯	◯	◯
Being chubby doesn’t equal unhealthy	◯	◯	◯	◯	◯
Being fat doesn’t equal unhealthy	◯	◯	◯	◯	◯
We should control the weight of our cats	◯	◯	◯	◯	◯
Being chubby says that the cat has a quality life	◯	◯	◯	◯	◯
Being fat says that the cat has a quality life	◯	◯	◯	◯	◯

31. If your cat is beside you now, can you please take a look and determine which set of the images best represent your cat's body shape?

- ◯ 1
- ◯ 2
- ◯ 3
- ◯ 4
- ◯ 5
- ◯ My cat is not around now

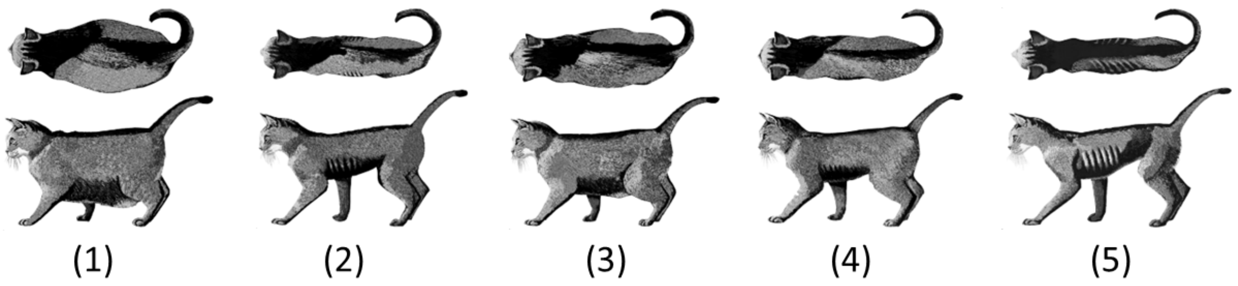

Australian Cat Body Composition Survey

Ownership

32. What was the main purpose for getting your cat?

- ◯ Companion for human(s)
- ◯ Companion for other animal(s)
- ◯ Pest control (e.g. rodents, cockroaches)
- ◯ Commercial related (e.g. cat café, attraction, etc.)
- ◯ Show cat
- ◯ The cat came to me so I feed/keep it
- ◯ No specific purpose
- ◯ Other (please specify)

33. What benefits have you received from having your cat apart from the main purpose? (select all that apply)

- ☐ Companion for human(s)
- ☐ Companion for other animal(s)
- ☐ Pest control (e.g. rodents, cockroaches)
- ☐ Commercial related (e.g. cat café, attraction, etc.)
- ☐ Show cat
- ☐ No other benefits

Other (please specify)

____

34. How often do you spend time with your cat doing each following?

	Never	Sometimes	Often	Always
Patting the cat	○	○	○	○
Playing with the cat	○	○	○	○
Doing my own things but enjoying the cat’s company in the same space	○	○	○	○

35. How satisfying is your relationship with your cat?

- ○ Not at all satisfying
- ○ Not very satisfying
- ○ Neutral
- ○ Satisfying
- ○ Very satisfying

36. How close are you to your cat?

- ○ Not at all close
- ○ Not very close
- ○ Neutral
- ○ Close
- ○ Very close

37. What are the top 2 reasons why you take your cat to a vet? (choose one for each column)

	1st reason	2nd reason
Health check	○	○
Vaccination	○	○
When something is wrong	○	○
Chronic illness	○	○
Deworm and flea treatment	○	○
Wash/ grooming/ nail cutting	○	○
Boarding	○	○

Australian Cat Body Composition Survey

Owner Demographics

38. What is your gender?

- ○ Female
- ○ Male
- ○ Other

39. What is your highest level of education?

- ○ Secondary school qualification
- ○ TAFE/ VET qualification or equivalent
- ○ Bachelor degree or equivalent
- ○ Master degree or equivalent
- ○ Doctoral degree or equivalent
- ○ Other (please specify)

40. What is the housing type of your place of residence?

- ○ Separate house
- ○ Semi-detached/ row or terrace house/ townhouse
- ○ Flat/ unit/ apartment
- ○ Other (please specify)

41. What is your residential household composition?

- ○ With your parent(s) and with/without sibling(s)
- ○ Shared household
- ○ Single person
- ○ Multiple families
- ○ One couple with dependent children
- ○ One-parent family with dependent children
- ○ One couple without dependent children
- ○ Other (please specify)

42. Have you ever lived in Australia for more than 10 years?

- ○ Yes, and I live in Australia now
- ○ Yes, but I don't live in Australia now
- ○ No

Australian Cat Body Composition Survey

Owner Demographics

43. What is the postcode of your place of residence?

44. Is your career/study animal related? (select all that apply)

- ☐ No
- ☐ Yes, veterinary profession related
- ☐ Yes, nonprofit animal organisation related
- ☐ Yes, companion animal related
- ☐ Yes, livestocks related
- ☐ Yes, wildlife related
- ☐ Yes, captive wildlife related
- ☐ Yes, other (please specify)

45. Where did you get the link of the questionnaire?

- ○ RSPCA
- ○ The University of Sydney
- ○ A veterinary clinic
- ○ Australian Cat Federation
- ○ SurveyMonkey
- ○ A cat show related webpage
- ○ Social media
- ○ An animal friendly restaurant/cafe
- ○ Other (please specify)

46. Please leave the preferred contact information so we can contact you if you win our $100 gift card lottery.

47. Are you willing to allow the researchers to contact you for future research purpose?

- ○ Yes
- ○ No

48. Please leave any comments you may have, thank you very much.
