# Supplementary figures and images for: Positive attitudes towards feline obesity are strongly associated with ownership of obese cats
Source: PLoS One. 2020 Jun 25;15(6):e0234190. doi: 10.1371/journal.pone.0234190 (PMC7316328; doi:10.1371/journal.pone.0234190)

## Participate in our survey and win a prize!

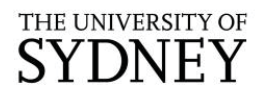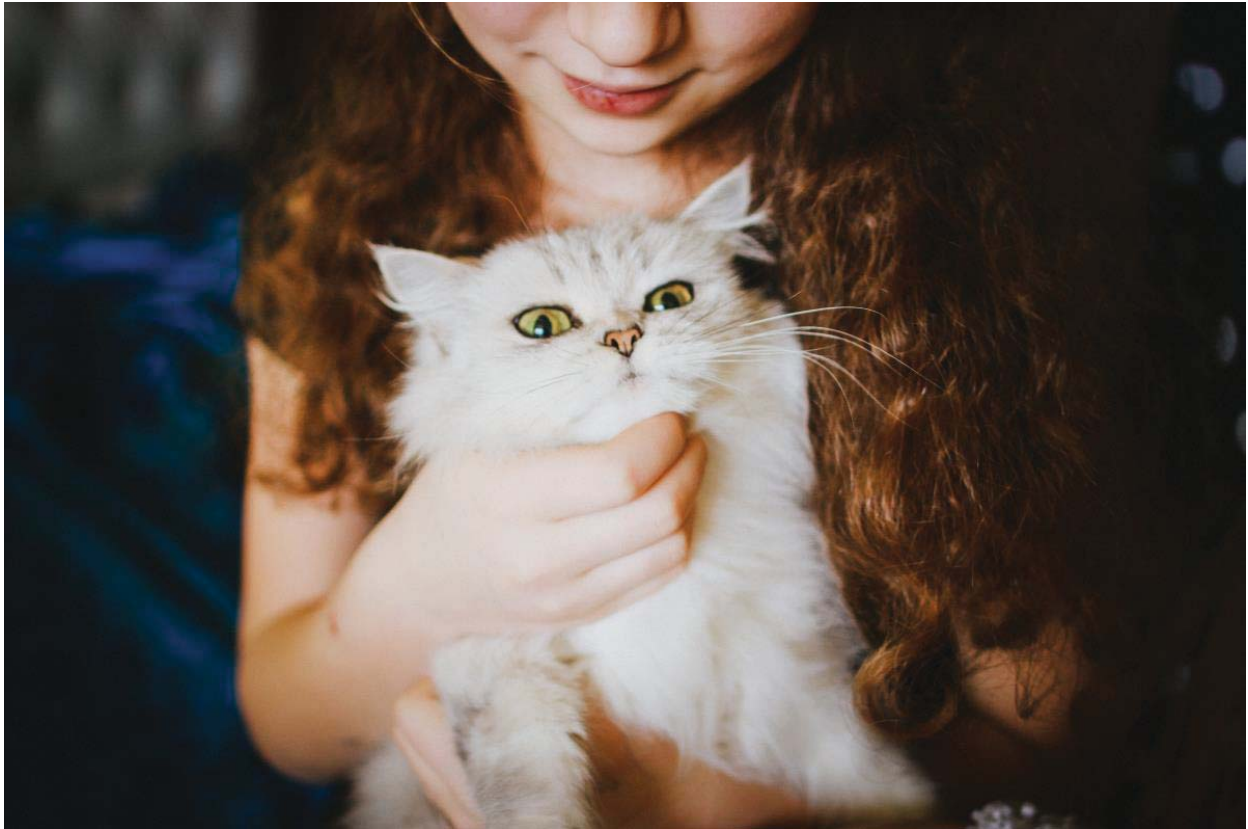[illegible]

Supplement: S2 File — (PDF) [file pone.0234190.s002.pdf]
