# Supplementary material for: Positive attitudes towards feline obesity are strongly associated with ownership of obese cats
Source: PLoS One. 2020 Jun 25;15(6):e0234190. doi: 10.1371/journal.pone.0234190 (PMC7316328; doi:10.1371/journal.pone.0234190)
Supplement: S1 Table — (DOCX) [file pone.0234190.s003.docx]

| **Risk factor** | **Source/s** |
| --- | --- |
| **Intrinsic** | |
| Male sex | Scarlett et al., 1994; Robertson, 1999; Lund et al., 2005; McGreevy et al., 2008; Colliard et al., 2009; Courcier et al., 2012; Öhlund et al., 2018 [1-7] |
| Neutered cats | Scarlett and Donoghue, 1996; Robertson, 1999; Russell et al., 2000; Lund et al., 2005; McGreevy et al., 2008; Colliard et al., 2009; Courcier et al., 2010; Öhlund et al., 2018 [2-4, 6-10] |
| Middle-aged cats | (Kronfeld et al., 1994; Scarlett et al., 1994; Scarlett and Donoghue, 1996; Lund et al., 2005; Kienzle and Berglert, 2006; Colliard et al., 2009; Teng et al., 2017; Öhlund et al., 2018 [1, 3, 4, 7, 8, 11-13] |
| Mixed-breed cats | Scarlett et al., 1994; Robertson, 1999; Lund et al., 2005; Teng et al., 2017; Öhlund et al., 2018 [1, 2, 4, 7, 13] |
| Long-haired cats | Colliard et al., 2009 [3] |
| Long leg length | Allan et al., 2000; Cave et al., 2012 [14, 15] |
| Faster growth rate between three and 12 months of age | [16] |
| **Extrinsic (positive association with feline O&O)** | |
| Feeding dry food | Rowe et al., 2015; Rowe et al., 2017 [17, 18] |
| Feeding premium or therapeutic dry food | Donoghue and Scarlett, 1998 [19] |
| Feeding premium or the therapeutic food (both dry and wet) | Lund et al., 2005 [4] |
| Feeding treats/table scraps | Russell et al., 2000; Kienzle and Berglert, 2006; Rowe et al., 2017 [9, 12, 18] |
| Feeding raw meat | Kienzle and Berglert, 2006 [12] |
| Feeding 2 to 3 times per day | Courcier et al., 2010 [10] |
| Feeding canned food *ad libitum* | Courcier et al., 2010 [10] |
| Food quantity fed determined by the recommendation on food packages | Colliard et al., 2009 [3] |
| Dogs present in household | Allan et al., 2000 [14] |
| Four or more cats present in household | Russell et al., 2000 [9] |
| Apartment dwelling | Russell et al., 2000 [9] |
| Veterinary clinic attended in a rural or semi-rural location | McGreevy et al., 2008 [6] |
| Non-illness related veterinary visit | Teng et al., 2017 [13] |
| **Extrinsic (negative association with feline O&O)** | |
| Owner plays with cat | Kienzle and Berglert, 2006 [12] |
| High activity level | Scarlett and Donoghue, 1996; Öhlund et al., 2018 [7, 8] |
| Outdoor access | Rowe et al., 2015; Teng et al., 2017 [13, 17] |
| Hunting activity by cat | Donoghue and Scarlett, 1998 [19] |

**Reference**

1. Scarlett JM, Donoghue S, Saidla J, Wills J. Overweight cats: prevalence and risk factors. Int J Obes. 1994;18:S22-S8. PubMed PMID: WOS:A1994NP87000004.

2. Robertson ID. The influence of diet and other factors on owner-perceived obesity in privately owned cats from metropolitan Perth, Western Australia. Prev Vet Med. 1999;40(2):75-85. doi: 10.1016/s0167-5877(99)00024-0. PubMed PMID: WOS:000080916700001.

3. Colliard L, Paragon BM, Lemuet B, Benet JJ, Blanchard G. Prevalence and risk factors of obesity in an urban population of healthy cats. J Feline Med Surg. 2009;11(2):135-40. doi: 10.1016/j.jfms.2008.07.002. PubMed PMID: WOS:000263499000012.

4. Lund EM, Armstrong PJ, Kirk CA, Klausner JS. Prevalence and risk factors for obesity in adult cats from private US veterinary practices. Int J Appl Res Vet Med. 2005;3(2):88-96.

5. Courcier E, Mellor D, Pendlebury E, Evans C, Yam P. An investigation into the epidemiology of feline obesity in Great Britain: results of a cross-sectional study of 47 companion animal practises. Veterinary Record. 2012;171(22):560-.

6. McGreevy P, Thomson P, Pride C, Fawcett A, Grassi T, Jones B. Overweight or obese cats presented to Australian veterinary practices, risk factors and prevalence. Aust Vet Pract. 2008;38(3):98-107. PubMed PMID: WOS:000260047800003.

7. Öhlund M, Palmgren M, Holst BS. Overweight in adult cats: a cross-sectional study. Acta Vet Scand. 2018;60(1):5. doi: 10.1186/s13028-018-0359-7.

8. Scarlett JM, Donoghue S. Obesity in cats: prevalence and prognosis. Veterinary Clinical Nutrition. 1996;3(4):128-32.

9. Russell K, Sabin R, Holt S, Bradley R, Harper EJ. Influence of feeding regimen on body condition in the cat. Journal of Small Animal Practice. 2000;41(1):12-7. doi: 10.1111/j.1748-5827.2000.tb03129.x. PubMed PMID: WOS:000084907400003.

10. Courcier EA, O'Higgins R, Mellor DJ, Yam PS. Prevalence and risk factors for feline obesity in a first opinion practice in Glasgow, Scotland. J Feline Med Surg. 2010;12(10):746-53. doi: 10.1016/j.jfms.2010.05.011. PubMed PMID: WOS:000282904300003.

11. Kronfeld D, Donoghue S, Glickman L. Body condition of cats. The Journal of Nutrition. 1994;124(12 Suppl):2683S-4S.

12. Kienzle E, Berglert R. Human-animal relationship of owners of normal and overweight cats. J Nutr. 2006;136(7):1947S-50S. PubMed PMID: WOS:000238753200006.

13. Teng KT, McGreevy PD, Toribio J-ALML, Raubenheimer D, Kendall K, Dhand NK. Risk factors for underweight and overweight in cats in metropolitan Sydney, Australia. Prev Vet Med. 2017;144:102-11. doi: <https://doi.org/10.1016/j.prevetmed.2017.05.021>.

14. Allan FJ, Pfeiffer DU, Jones BR, Esslemont DHB, Wiseman MS. A cross-sectional study of risk factors for obesity in cats in New Zealand. Prev Vet Med. 2000;46(s3):183-96. doi: 10.1016/s0167-5877(00)00147-1. PubMed PMID: WOS:000088663800004.

15. Cave NJ, Allan FJ, Schokkenbroek SL, Metekohy CAM, Pfeiffer DU. A cross-sectional study to compare changes in the prevalence and risk factors for feline obesity between 1993 and 2007 in New Zealand. Prev Vet Med. 2012;107(1-2):121-33. doi: 10.1016/j.prevetmed.2012.05.006. PubMed PMID: WOS:000310040600013.

16. Serisier S, Feugier A, Venet C, Biourge V, German AJ. Faster growth rate in ad libitum-fed cats: a risk factor predicting the likelihood of becoming overweight during adulthood. J Nutr Sci. 2013;2:e11. doi: doi:10.1017/jns.2013.10.

17. Rowe E, Browne W, Casey R, Gruffydd-Jones T, Murray J. Risk factors identified for owner-reported feline obesity at around one year of age: Dry diet and indoor lifestyle. Prev Vet Med. 2015;121(3–4):273-81. doi: <http://dx.doi.org/10.1016/j.prevetmed.2015.07.011>.

18. Rowe EC, Browne WJ, Casey RA, Gruffydd-Jones TJ, Murray JK. Early-life risk factors identified for owner-reported feline overweight and obesity at around two years of age. Prev Vet Med. 2017;143(Supplement C):39-48. doi: <https://doi.org/10.1016/j.prevetmed.2017.05.010>.

19. Donoghue S, Scarlett JM. Diet and feline obesity. The Journal of Nutrition. 1998;128(12):2776S-8S.
