# Supplementary material for: Positive attitudes towards feline obesity are strongly associated with ownership of obese cats
Source: PLoS One. 2020 Jun 25;15(6):e0234190. doi: 10.1371/journal.pone.0234190 (PMC7316328; doi:10.1371/journal.pone.0234190)
Supplement: S2 Table — (DOCX) [file pone.0234190.s004.docx]

| **Variable** | **Category** | **BCS1** | **BCS2** | **BCS3** | **BCS4** | **BCS5** | **Total** | **Grand total** |
| --- | --- | --- | --- | --- | --- | --- | --- | --- |
| Daily feeding frequency | 1/day | 2 (1.1%) | 10 (5.7%) | 133 (76.0%) | 28 (16.0%) | 2 (1.1%) | 175 (12.7%) | 1,379 (99.2%) |
|  | 2/day | 3 (0.4%) | 43 (5.1%) | 583 (69.3%) | 191 (22.7%) | 21 (2.5%) | 841 (61.0%) |  |
|  | 3/day | 2 (1.2%) | 14 (8.6%) | 112 (68.7%) | 33 (20.2%) | 2 (1.2%) | 163 (11.8%) |  |
|  | ≥4/day | 2 (3.8%) | 8 (15.4%) | 27 (51.9%) | 14 (26.9%) | 1 (1.9%) | 52 (3.8%) |  |
|  | Ad libitum | 2 (1.4%) | 14 (9.5%) | 93 (62.8%) | 36 (24.3%) | 3 (2.0%) | 148 (10.7%) |  |
| Dry food^1^ | Never | 1 (2.7%) | 7 (18.92) | 18 (48.6%) | 10 (27.0%) | 1 (2.7%) | 37 (2.7%) | 1,368 (98.4%) |
|  | Minor diet | 3 (0.7%) | 39 (8.7%) | 320 (71.7%) | 76 (17.0%) | 8 (1.8%) | 446 (32.6%) |  |
|  | Major diet | 5 (0.7%) | 37 (4.9%) | 516 (68.4%) | 178 (23.6%) | 18 (2.4%) | 754 (55.1%) |  |
|  | Only diet | 1 (0.8%) | 6 (4.6%) | 86 (65.6%) | 36 (27.5%) | 2 (1.5%) | 131 (9.6%) |  |
| Canned food^1^ | Never | 2 (0.5%) | 24 (5.5%) | 307 (70.1%) | 98 (22.4%) | 7 (1.6%) | 438 (35.4%) | 1,236 (88.9%) |
|  | Minor diet | 4 (0.9%) | 27 (6.4%) | 281 (66.3%) | 102 (24.1%) | 10 (2.4%) | 424 (34.3%) |  |
|  | Major diet | 3 (0.8%) | 28 (7.6%) | 258 (70.1%) | 72 (19.6%) | 7 (1.9%) | 368 (29.8%) |  |
|  | Only diet | 0 (0.0%) | 2 (33.3%) | 4 (66.7%) | 0 (0.0%) | 0 (0.0%) | 6 (0.5%) |  |
| Wet food apart from cans^1^ | Never | 6 (1.5%) | 24 (5.8%) | 278 (67.5%) | 99 (24.03%) | 5 (1.2%) | 412 (33.2%) | 1,242 (89.4%) |
|  | Minor diet | 2 (0.4%) | 27 (5.8%) | 323 (69.6%) | 101 (21.8%) | 11 (2.4%) | 464 (37.4%) |  |
|  | Major diet | 1 (0.3%) | 30 (8.4%) | 259 (72.3%) | 62 (17.3%) | 6 (1.7%) | 358 (28.8%) |  |
|  | Only diet | 1 (12.5%) | 2 (25.0%) | 3 (37.5%) | 2 (25.0%) | 0 (0.0%) | 8 (0.6%) |  |
| Home-made cat food^1^ | Never | 7 (0.7%) | 58 (6.2%) | 653 (69.5%) | 206 (21.9%) | 15 (1.6%) | 939 (81.0%) | 1,159 (83.4%) |
|  | Minor diet | 1 (0.7%) | 11 (7.7%) | 99 (69.2%) | 27 (18.9%) | 5 (3.5%) | 143 (12.3%) |  |
|  | Major diet | 0 (0.0%) | 5 (7.4%) | 49 (72.1%) | 12 (17.6%) | 2 (2.9%) | 68 (5.9%) |  |
|  | Only diet | 1 (11.1%) | 0 (0.0%) | 6 (66.7%) | 2 (22.2%) | 0 (0.0%) | 9 (0.8%) |  |
| Leftovers of human food^1^ | Never | 6 (0.8%) | 46 (5.8%) | 553 (70.3%) | 167 (21.2%) | 15 (1.9%) | 787 (67.7%) | 1,163 (83.7%) |
|  | Minor diet | 3 (0.8%) | 32 (8.7%) | 249 (67.7%) | 78 (21.2%) | 6 (1.6%) | 368 (31.6%) |  |
|  | Major diet | 0 (0.0%) | 0 (0.0%) | 4 (66.7%) | 2 (33.3%) | 0 (0.0%) | 6 (0.5%) |  |
|  | Only diet | 0 (0.0%) | 0 (0.0%) | 1 (50.0%) | 1 (50.0%) | 0 (0.0%) | 2 (0.2%) |  |
|  |  |  |  |  |  |  |  |  |
| **Variable** | **Category** | **BCS1** | **BCS2** | **BCS3** | **BCS4** | **BCS5** | **Total** | **Grand total** |
| Treats/ snacks^2^ | Never | 5 (1.0%) | 33 (6.5%) | 343 (67.1%) | 118 (23.1%) | 12 (2.3%) | 511 (42.7%) | 1,197 (86.1%) |
|  | Minor diet | 5 (0.7%) | 43 (6.3%) | 484 (71.4%) | 135 (19.9%) | 11 (1.6%) | 678 (56.6%) |  |
|  | Major diet | 0 (0.0%) | 0 (0.0%) | 5 (71.4%) | 2 (28.6%) | 0 (0.0%) | 7 (0.6%) |  |
|  | Only diet | 0 (0.0%) | 0 (0.0%) | 0 (0.0%) | 1 (100.0%) | 0 (0.0%) | 1 (0.1%) |  |
| Quantity: no specific rules^3^ | No | 9 (0.8%) | 78 (7.3%) | 733 (68.2%) | 228 (21.2%) | 26 (2.4%) | 1,074 (78.2%) | 1,374 (98.8%) |
|  | Yes | 2 (0.7%) | 10 (3.3%) | 212 (70.7%) | 73 (24.3%) | 3 (1.0%) | 300 (21.8%) |  |
| Quantity: advice from veterinarians^3^ | No | 8 (0.8%) | 59 (5.9%) | 708 (70.3%) | 215 (21.4%) | 17 (1.7%) | 1,007 (73.3%) | 1,374 (98.8%) |
|  | Yes | 3 (0.8%) | 29 (7.9%) | 237 (64.6%) | 86 (23.4%) | 12 (3.3%) | 367 (26.7%) |  |
| Quantity: advice from the package^3^ | No | 7 (0.7%) | 78 (7.9%) | 674 (68.6%) | 205 (20.9%) | 19 (1.9%) | 983 (71.5%) | 1,374 (98.8%) |
|  | Yes | 4 (1.0%) | 10 (2.6%) | 271 (69.3%) | 96 (24.6%) | 10 (2.6%) | 391 (28.5%) |  |
| Quantity: the amount that the cat ate^3^ | No | 6 (0.6%) | 42 (4.5%) | 632 (67.2%) | 239 (25.4%) | 22 (2.3%) | 941 (68.5%) | 1,374 (98.8%) |
|  | Yes | 5 (1.2%) | 46 (10.6%) | 313 (72.3%) | 62 (14.3%) | 7 (1.6%) | 433 (31.5%) |  |
| Quantity: more than my cat needs^3^ | No | 9 (0.7%) | 84 (6.4%) | 915 (69.5%) | 282 (21.4%) | 26 (2.0%) | 1,316 (95.8%) | 1,374 (98.8%) |
|  | Yes | 2 (3.4%) | 4 (6.9%) | 30 (51.7%) | 19 (32.8%) | 3 (5.2%) | 58 (4.2%) |  |
| Quantity: based on body weight^3^ | No | 11 (0.9%) | 76 (6.5%) | 788 (67.9%) | 260 (22.4%) | 26 (2.2%) | 1,161 (84.5%) | 1,374 (98.8%) |
|  | Yes | 0 (0.0%) | 12 (5.6%) | 157 (73.7%) | 41 (19.2%) | 3 (1.4%) | 213 (15.5%) |  |
| Quantity: based on BCS^3^ | No | 11 (1%) | 64 (5.8%) | 753 (68.5%) | 245 (22.3%) | 27 (2.5%) | 1,100 (80.1%) | 1,374 (98.8%) |
|  | Yes | 0 (0.0%) | 24 (8.8%) | 192 (70.1%) | 56 (20.4%) | 2 (0.7%) | 274 (19.9%) |  |
| Quantity: other^3^ | No | 11 (0.8%) | 87 (6.5%) | 917 (68.8%) | 290 (21.8%) | 27 (2.0%) | 1332 (96.9%) | 1,374 (98.8%) |
|  | Yes | 0 (0.0%) | 1 (2.4%) | 28 (66.7%) | 11 (26.2%) | 2 (4.8%) | 42 (3.1%) |  |
| Leftover of human food | Never | 1 (0.3%) | 19 (5.0%) | 251 (65.5%) | 101 (26.4%) | 11 (2.9%) | 383 (27.8%) | 1,376 (99.0%) |
|  | Sometimes | 3 (0.4%) | 45 (6.7%) | 472 (70.0%) | 140 (20.8%) | 14 (2.1%) | 674 (49.0%) |  |
|  | Often | 4 (1.9%) | 19 (9.1%) | 145 (69.7%) | 38 (18.3%) | 2 (1.0%) | 208 (15.1%) |  |
|  | Always | 3 (2.7%) | 5 (4.5%) | 78 (70.3%) | 23 (20.7%) | 2 (1.8%) | 111 (8.1%) |  |
| **Variable** | **Category** | **BCS1** | **BCS2** | **BCS3** | **BCS4** | **BCS5** | **Total** | **Grand total** |
| Food begging behaviours | Never | 2 (0.6%) | 19 (5.5%) | 266 (77.6%) | 51 (14.9%) | 5 (1.5%) | 343 (24.9%) | 1,377 (99.1%) |
|  | Sometimes | 5 (0.7%) | 50 (6.7%) | 527 (70.8%) | 150 (20.2%) | 12 (1.6%) | 744 (54.0%) |  |
|  | Often | 2 (1.1%) | 12 (6.3%) | 106 (56.1%) | 61 (32.3%) | 8 (4.2%) | 189 (13.7%) |  |
|  | Always | 2 (2.0%) | 8 (7.9%) | 47 (46.5%) | 40 (39.6%) | 4 (4.0%) | 101 (7.3%) |  |
| Owner giving in to begging | Never | 1 (0.2%) | 20 (4.5%) | 335 (74.8%) | 81 (18.1%) | 11 (2.5%) | 448 (32.7%) | 1,372 (98.7%) |
|  | Sometimes | 5 (0.8%) | 36 (5.5%) | 444 (67.6%) | 160 (24.4%) | 12 (1.8%) | 657 (47.9%) |  |
|  | Often | 1 (0.6%) | 19 (11.4%) | 95 (56.9%) | 47 (28.1%) | 5 (3.0%) | 167 (12.2%) |  |
|  | Always | 4 (4.0%) | 12 (12.0%) | 69 (69.0%) | 14 (1.0%) | 1 (1.0%) | 100 (7.3%) |  |
| Food sources other than the owner^4^ | Never | 10 (0.9%) | 74 (6.6%) | 772 (68.6%) | 245 (21.8%) | 24 (2.1%) | 1,125 (81.6%) | 1,379 (99.2%) |
|  | Probably not | 1 (0.6%) | 11 (6.5%) | 122 (72.2%) | 31 (18.3%) | 4 (2.4%) | 169 (12.3%) |  |
|  | Often | 0 (0.0%) | 0 (0.0%) | 11 (84.6%) | 2 (15.4%) | 0 (0.0%) | 13 (0.9%) |  |
|  | Probably yes | 0 (0.0%) | 4 (6.1%) | 41 (62.1%) | 20 (30.3%) | 1 (1.5%) | 66 (4.8%) |  |
|  | All the time | 0 (0.0%) | 0 (0.0%) | 2 (33.3%) | 4 (66.7%) | 0 (0.0%) | 6 (0.4%) |  |

^1^: For these variables, feeding frequency categories ‘major diet’ and ‘only diet’ were combined into ‘major diet’ for statistical analyses.

^2^: The feeding frequency of *leftovers of human food* and *treats/snacks* were re-categorised into feeding (‘yes’) or not feeding (‘no’) for statistical analyses.

^3^: Each method used to determine the quantity of food was transformed into binary variables (‘yes’ and ‘no’).

^4^: The frequency of getting food from outside resource was grouped into ‘yes’ (including options ‘all the time’, ‘often’ and ‘I am not sure but probably yes’) and ‘no’ (including options ‘never’ and ‘I am not sure but probably not’) for statistical analyses.
