# Supplementary material for: Positive attitudes towards feline obesity are strongly associated with ownership of obese cats
Source: PLoS One. 2020 Jun 25;15(6):e0234190. doi: 10.1371/journal.pone.0234190 (PMC7316328; doi:10.1371/journal.pone.0234190)
Supplement: S3 Table — (DOCX) [file pone.0234190.s005.docx]

| **Variable** | **Category** | | **BCS1** | | | **BCS2** | | **BCS3** | | | **BCS4** | | | **BCS5** | | **Total** | | | | **Grand total** | | |
| --- | --- | --- | --- | --- | --- | --- | --- | --- | --- | --- | --- | --- | --- | --- | --- | --- | --- | --- | --- | --- | --- | --- |
| Stay indoors^1^ | I am not sure | | 0 (0.0%) | | | 0 (0.0%) | | 0 (0.0%) | | | 1 (100.0%) | | | 0 (0.0%) | | 1 (0.1%) | | | | 1351 (97.2%) | | |
|  | Never | | 0 (0.0%) | | | 0 (0.0%) | | 7 (100.0%) | | | 0 (0.0%) | | | 0 (0.0%) | | 7 (0.5%) | | | |  |  |  |
|  | Sometimes | | 0 (0.0%) | | | 4 (6.7%) | | 40 (66.7%) | | | 14 (23.3%) | | | 2 (3.3%) | | 60 (4.4%) | | | |  | | |
|  | Often | | 3 (0.5%) | | | 34 (6.0%) | | 391 (69.6%) | | | 122 (21.7%) | | | 12 (2.1%) | | 562 (41.6%) | | | |  | | |
|  | Always | | 7 (1.0%) | | | 48 (6.7%) | | 492 (68.2%) | | | 159 (22.1%) | | | 15 (2.1%) | | 721 (53.4%) | | | |  | | |
| Staying outdoors but still inside the property^1^ | I am not sure | | 0 (0.0%) | | | 0 (0.0%) | | 0 (0.0%) | | | 1 (100.0%) | | | 0 (0.0%) | | 1 (0.1%) | | | | 1,310 (94.2%) | | |
|  | Never | | 4 (1.0%) | | | 23 (5.8%) | | 263 (66.4%) | | | 96 (24.2%) | | | 10 (2.5%) | | 396 (30.2%) | | | |  |  |  |
|  | Sometimes | | 3 (0.7%) | | | 34 (7.4%) | | 311 (68.1%) | | | 101 (22.1%) | | | 8 (1.8%) | | 457 (34.9%) | | | |  | | |
|  | Often | | 2 (0.5%) | | | 26 (6.4%) | | 292 (71.9%) | | | 77 (19.0%) | | | 9 (2.2%) | | 406 (31.0%) | | | |  | | |
|  | Always | | 0 (0.0%) | | | 0 (0.0%) | | 39 (78.0%) | | | 9 (18.0%) | | | 2 (4.0%) | | 50 (3.8%) | | | |  | | |
| Stay outdoors but outside the property^1^ | I am not sure | | 0 (0.0%) | | | 5 (9.8%) | | 34 (66.7%) | | | 10 (19.6%) | | | 2 (3.9%) | | 51 (4.0%) | | | | 1,263 (90.9%) | | |
|  | Never | | 7 (0.8%) | | | 56 (6.3%) | | 601 (67.8%) | | | 200 (22.5%) | | | 23 (2.6%) | | 887 (70.2%) | | | |  |  |  |
|  | Sometimes | | 1 (0.4%) | | | 16 (6.3%) | | 182 (71.4%) | | | 54 (21.2%) | | | 2 (0.8%) | | 255 (20.2%) | | | |  | | |
|  | Often | | 0 (0.0%) | | | 4 (6.0%) | | 54 (80.6%) | | | 9 (13.4%) | | | 0 (0.0%) | | 67 (5.3%) | | | |  | | |
|  | Always | | 0 (0.0%) | | | 0 (0.0%) | | 3 (100.0%) | | | 0 (0.0%) | | | 0 (0.0%) | | 3 (0.2%) | | | |  | | |
| Hunting frequency^2^ | I am not sure | | 0 (0.0%) | | | 5 (6.8%) | | 48 (65.8%) | | | 19 (26.0%) | | | 1 (1.4%) | | 73 (5.4%) | | | | 1,352 (97.3%) | | |
|  | Never | | 8 (1.2%) | | | 49 (7.1%) | | 455 (66.3%) | | | 159 (23.2%) | | | 15 (2.2%) | | 686 (50.7%) | | | |  |  |  |
|  | Rarely | | 2 (0.7%) | | | 20 (6.9%) | | 201 (69.3%) | | | 57 (19.7%) | | | 10 (3.4%) | | 290 (21.4%) | | | |  |  |  |
|  | Sometimes | | 0 (0.0%) | | | 7 (4.5%) | | 110 (70.1%) | | | 37 (23.6%) | | | 3 (1.9%) | | 157 (11.6%) | | | |  | | |
|  | Often | | 0 (0.0%) | | | 4 (4.8%) | | 68 (81.0%) | | | 12 (14.3%) | | | 0 (0.0%) | | 84 (6.2%) | | | |  | | |
|  | Always | | 0 (0.0%) | | | 1 (1.6%) | | 49 (79.0%) | | | 12 (19.4%) | | | 0 (0.0%) | | 62 (4.6%) | | | |  | | |
| Prey: bird | No | | 9 (0.8%) | | | 77 (6.4%) | | 820 (68.3%) | | | 269 (22.4%) | | | 25 (2.1%) | | 1,200 (88.7%) | | | | 1,353 (97.3%) | | |
|  | Yes | | 1 (0.7%) | | | 9 (5.9%) | | 112 (73.2%) | | | 27 (17.6%) | | | 4 (2.6%) | | 153 (11.3%) | | | |  |  |  |
| Prey: larger mammal | No | | 10 (0.8%) | | | 86 (6.5%) | | 914 (68.8%) | | | 290 (21.8%) | | | 29 (2.2%) | | 1,329 (98.2%) | | | | 1,353 (97.3%) | | |
|  | Yes | | 0 (0.0%) | | | 0 (0.0%) | | 18 (75.0%) | | | 6 (25.0%) | | | 0 (0.0%) | | 24 (1.8%) | | | |  |  |  |
| **Variable** | **Category** | | **BCS1** | | | **BCS2** | | **BCS3** | | | **BCS4** | | | **BCS5** | | **Total** | | | | **Grand total** | | |
| Prey: small mammal | No | | 8 (0.7%) | | | 78 (6.9%) | | 775 (68.2%) | | | 249 (21.9%) | | | 26 (2.3%) | | 1,136 (84.0%) | | | | 1,353 (97.3%) | | |
|  | Yes | | 2 (0.9%) | | | 8 (3.7%) | | 157 (72.4%) | | | 47 (21.7%) | | | 3 (1.4%) | | 217 (16.0%) | | | |  |  |  |
| Prey: insect | No | | 10 (1.1%) | | | 57 (6.5%) | | 597 (68.2%) | | | 192 (21.9%) | | | 19 (2.2%) | | 875 (64.7%) | | | | 1,353 (97.3%) | | |
|  | Yes | | 0 (0.0%) | | | 29 (6.1%) | | 335 (70.1%) | | | 104 (21.8%) | | | 10 (2.1%) | | 478 (35.3%) | | | |  |  |  |
| Prey: lizard and frog | No | | 10 (0.8%) | | | 75 (6.3%) | | 819 (69.2%) | | | 255 (21.5%) | | | 25 (2.1%) | | 1,184 (87.5%) | | | | 1,353 (97.3%) | | |
|  | Yes | | 0 (0.0%) | | | 11 (6.5%) | | 113 (66.9%) | | | 41 (24.3%) | | | 4 (2.4%) | | 169 (12.5%) | | | |  |  |  |
| Prey type^3^ | Large animals^4^ | | 0 (0.0%) | | 2 (7.4%) | | | 17 (63.0%) | | 5 (18.5%) | | | 3 (11.1%) | | | | 27 (2.0%) | | 1,353 (97.3%) | | |  |
|  | Small animals^5^ | | 1 (0.2%) | | 28 (5.2%) | | | 382 (70.9%) | | 118 (21.9%) | | | 10 (1.9%) | | | | 539 (39.8%) | |  |  |  |  |
|  | Both large and small animals | | 1 (0.7%) | | 7 (5.1%) | | | 103 (75.7%) | | 24 (17.6%) | | | 1 (0.7%) | | | | 136 (10.1%) | |  | | |  |
|  | No hunting | | 8 (1.2%) | | 49 (7.5%) | | | 430 (66.1%) | | 149 (22.9%) | | | 15 (2.3%) | | | | 651 (48.1%)^1^ | |  | | |  |
| Going out at night^6^ | I am not sure | | 0 (0.0%) | | 0 (0.0%) | | | 9 (69.2%) | | 4 (30.8%) | | | 0 (0.0%) | | | | 13 (1.0%) | | 1,351 (97.2%) | | |  |
|  | Never | | 7 (0.7%) | | 70 (6.8%) | | | 719 (69.5%) | | 217 (21.0%) | | | 22 (2.1%) | | | | 1,035 (76.6%) | |  |  |  |  |
|  | Rarely | | 0 (0.0%) | | 7 (7.7%) | | | 59 (64.8%) | | 23 (25.3%) | | | 2 (2.2%) | | | | 91 (6.7%) | |  | | |  |
|  | Occasionally | | 0 (0.0%) | | 4 (9.1%) | | | 26 (59.1%) | | 12 (27.3%) | | | 2 (4.5%) | | | | 44 (3.3%) | |  | | |  |
|  | Quite often | | 2 (3.6%) | | 1 (1.8%) | | | 42 (76.4%) | | 10 (18.2%) | | | 0 (0.0%) | | | | 55 (4.1%) | |  | | |  |
|  | Always | | 1 (0.9%) | | 3 (2.7%) | | | 76 (67.3%) | | 30 (26.5%) | | | 3 (2.7%) | | | | 113 (8.4%) | |  | | |  |
| Owner patting the cat^7^ | Never | | 0 (0.0%) | | 1 (25.0%) | | | 3 (75.0%) | | 0 (0.0%) | | | 0 (0.0%) | | | | 4 (0.3%) | | 1,345 (96.8%) | | |  |
|  | Sometimes | | 0 (0.0%) | | 7 (7.7%) | | | 65 (71.4%) | | 17 (18.7%) | | | 2 (2.2%) | | | | 91 (6.8%) | |  |  |  |  |
|  | Often | | 6 (0.7%) | | 54 (6.3%) | | | 588 (68.7%) | | 191 (22.3%) | | | 17 (2.0%) | | | | 856 (63.6%) | |  | | |  |
|  | Always | | 4 (1.0%) | | 25 (6.3%) | | | 269 (68.3%) | | 87 (22.1%) | | | 10 (2.5%) | | | | 394 (29.3%) | |  | | |  |
| Owner playing with the cat^7^ | Never | | 1 (3.0%) | | 4 (12.1%) | | | 21 (63.6%) | | 7 (21.2%) | | | 0 (0.0%) | | | | 33 (2.5%) | | 1,344 (96.7%) | | |  |
|  | Sometimes | | 4 (0.8%) | | 36 (7.5%) | | | 311 (64.4%) | | 114 (23.6%) | | | 18 (3.7%) | | | | 483 (35.9%) | |  |  |  |  |
|  | Often | | 4 (0.6%) | | 38 (5.7%) | | | 479 (71.7%) | | 137 (20.5%) | | | 10 (1.5%) | | | | 668 (49.7%) | |  |  |  |  |
|  | Always | | 1 (0.6%) | | 8 (5.0%) | | | 114 (71.3%) | | 36 (22.5%) | | | 1 (0.6%) | | | | 160 (11.9%) | |  | | |  |
| **Variable** | **Category** | **BCS1** | | **BCS2** | | | **BCS3** | | **BCS4** | | | **BCS5** | | | **Total** | | | **Grand total** | | |  |  |
| Owner doing his/her own things with the cat’s company^7^ | Never | | 0 (0.0%) | | 1 (16.7%) | | | 4 (66.7%) | | 1 (16.7%) | | | 0 (0.0%) | | | | 6 (0.4%) | | 1,343 (96.7%) | | |  |
|  | Sometimes | | 0 (0.0%) | | 3 (4.1%) | | | 54 (74.0%) | | 14 (19.2%) | | | 2 (2.7%) | | | | 73 (5.4%) | |  |  |  |  |
|  | Often | | 5 (0.7%) | | 45 (6.4%) | | | 478 (68.3%) | | 155 (22.1%) | | | 17 (2.4%) | | | | 700 (52.1%) | |  | | |  |
|  | Always | | 5 (0.9%) | | 37 (6.6%) | | | 389 (69.0%) | | 123 (21.8%) | | | 10 (1.8%) | | | | 564 (42.0%) | |  | | |  |

^1^: How often the cats *stay indoors*, *outdoors in the property*, and *outdoors outside the property*, respectively, was categorised into ‘not often’ (including options ‘never’ and ‘sometimes’) and ‘often’ (including options ‘often’ and ‘always’) for statistical analyses.

^2^: *Hunting frequency* were regrouped into three categories: ‘never’, ‘sometimes’ (including options ‘rarely (≤once a month)’ and ‘sometimes (≤once a month)’), and ‘often’ (including options ‘often (≤once a week)’ and ‘always (≤once every 2–3 days)’) for statistical analyses.

^3^: *Prey type* had three levels in statistical analyses; ‘large animals’ included birds or large mammals (e.g., rabbits, squirrels, possums), and otherwise a hunting cat was a ‘small animals’ hunter. Cats with no prey specified belonged to ‘not hunting’

^4^: Birds and large mammals

^5^: Small mammals, insects, lizards and frogs

^6^: The frequency of the cat *going out at night* was regrouped using the same way to ‘*hunting frequency*’ for statistical analyses.

^7^: Three variables related to the interactions between the participants and their cats (*patting the cat, playing with the cat, doing my own things with the cat’s company*) were regrouped into ‘not often’ (options ‘never’ and ‘sometimes’), and ‘often’ and ‘always’ for statistical analyses.
