# Supplementary material for: Positive attitudes towards feline obesity are strongly associated with ownership of obese cats
Source: PLoS One. 2020 Jun 25;15(6):e0234190. doi: 10.1371/journal.pone.0234190 (PMC7316328; doi:10.1371/journal.pone.0234190)
Supplement: S4 Table — (DOCX) [file pone.0234190.s006.docx]

| **Variable** | **Category** | **BCS1** | **BCS2** | **BCS3** | **BCS4** | **BCS5** | **Total** | **Grand total** |
| --- | --- | --- | --- | --- | --- | --- | --- | --- |
| Number of cats | 1 | 7 (1.0%) | 50 (7.3%) | 468 (68.4%) | 147 (21.5%) | 12 (1.8%) | 684 (49.2%) | 1,389 (99.9%) |
|  | 2 | 4 (0.9%) | 25 (5.6%) | 300 (66.7%) | 112 (24.9%) | 9 (2.0%) | 450 (32.4%) |  |
|  | ≥3 | 0 (0.0%) | 14 (5.5%) | 185 (72.5%) | 47 (18.4%) | 9 (3.5%) | 255 (18.4%) |  |
| Gender^1^ | Female | 7 (0.6%) | 76 (6.4%) | 815 (68.7%) | 260 (21.9%) | 28 (2.4%) | 1186 (89.0%) | 1333 (95.9%) |
|  | Male | 2 (1.4%) | 9 (6.2%) | 101 (69.7%) | 32 (22.1%) | 1 (0.7%) | 145 (10.8%) |  |
|  | Other | 0 (0.0%) | 0 (0.0%) | 2 (100%) | 0 (0.0%) | 0 (0.0%) | 2 (0.2%) |  |
| Age range | 18–24 | 0 (0.0%) | 13 (7.0%) | 138 (73.8%) | 35 (18.7%) | 1 (0.5%) | 187 (13.5%) | 1390 (100%) |
|  | 25–34 | 1 (0.2%) | 17 (4.2%) | 285 (70.4%) | 94 (23.2%) | 8 (2%) | 405 (29.1%) |  |
|  | 35–44 | 8 (2.5%) | 22 (7.0%) | 204 (64.8%) | 70 (22.2%) | 11 (3.5%) | 315 (22.7%) |  |
|  | 45–54 | 2 (0.8%) | 20 (7.5%) | 180 (67.7%) | 60 (22.6%) | 4 (1.5%) | 266 (19.1%) |  |
|  | 55–64 | 0 (0.0%) | 14 (8.9%) | 106 (67.5%) | 32 (20.4%) | 5 (3.2%) | 157 (11.3%) |  |
|  | 65 and above | 0 (0.0%) | 3 (5.0%) | 41 (68.3%) | 15 (25.0%) | 1 (1.7%) | 60 (4.3%) |  |
| Education level | Secondary school qualification | 1 (0.4%) | 10 (4.0%) | 185 (74.6%) | 49 (19.8%) | 3 (1.2%) | 248 (18.6%) | 1330 (95.7%) |
|  | TAFE^2^/VET^3^ qualification^4^ | 4 (1.2%) | 24 (7.0%) | 236 (68.6%) | 73 (21.2%) | 7 (2.0%) | 344 (25.9%) |  |
|  | Bachelors degree^4^ | 2 (0.4%) | 32 (6.3%) | 343 (67.7%) | 119 (23.5%) | 11 (2.2%) | 507 (38.1%) |  |
|  | Masters degree^4^ | 2 (1.2%) | 11 (6.5%) | 116 (69.0%) | 33 (19.6%) | 6 (3.6%) | 168 (12.6%) |  |
|  | Doctoral degree^4^ | 0 (0.0%) | 3 (6.8%) | 29 (65.9%) | 10 (22.7%) | 2 (4.5%) | 44 (3.3%) |  |
|  | Other | 0 (0.0%) | 5 (26.3%) | 8 (42.1%) | 6 (31.6%) | 0 (0.0%) | 19 (1.4%) |  |
| Being a veterinarian | No | 10 (0.8%) | 81 (6.7%) | 844 (69.6%) | 253 (20.9%) | 24 (2.0%) | 1212 (93%) | 1303 (93.7%) |
|  | Yes | 0 (0.0%) | 1 (1.1%) | 55 (60.4%) | 30 (33.0%) | 5 (5.5%) | 91 (7.0%) |  |
| Animal-related profession | No | 9 (0.8%) | 75 (6.8%) | 764 (69.2%) | 236 (21.4%) | 20 (1.8%) | 1104 (84.7%) | 1303 (93.7%) |
|  | Yes | 1 (0.5%) | 7 (3.5%) | 135 (67.8%) | 47 (23.6%) | 9 (4.5%) | 199 (15.3%) |  |
| Dwelling type | Flat | 2 (0.7%) | 15 (5.6%) | 175 (64.8%) | 71 (26.3%) | 7 (2.6%) | 270 (20.3%) | 1,329 (95.6%) |
|  | Townhouse | 1 (0.6%) | 17 (10.3%) | 96 (58.2%) | 45 (27.3%) | 6 (3.6%) | 165 (12.4%) |  |
|  | House | 6 (0.7%) | 53 (5.9%) | 645 (72.1%) | 174 (19.5%) | 16 (1.8%) | 894 (67.3%) |  |
| **Variable** | **Category** | **BCS1** | **BCS2** | **BCS3** | **BCS4** | **BCS5** | **Total** | **Grand total** |
| Household | Single person | 4 (1.6%) | 22 (8.9%) | 157 (63.3%) | 58 (23.4%) | 7 (2.8%) | 248 (18.6%) | 1332 (95.8%) |
|  | Shared household | 0 (0.0%) | 10 (8.3%) | 83 (68.6%) | 23 (19.0%) | 5 (4.1%) | 121 (9.1%) |  |
|  | Family | 5 (0.5%) | 53 (5.5%) | 677 (70.3%) | 211 (21.9%) | 17 (1.2%) | 963 (72.3%) |  |

^1^: *Gender* excluded participants who were neither male nor female for statistical analyses.

^2^: Technical and further education

^3^: Vocational education and training

^4^: Or equivalent
