# Supplementary material for: Positive attitudes towards feline obesity are strongly associated with ownership of obese cats
Source: PLoS One. 2020 Jun 25;15(6):e0234190. doi: 10.1371/journal.pone.0234190 (PMC7316328; doi:10.1371/journal.pone.0234190)
Supplement: Attachment — Submitted filename: rebuttal_20200415.docx [file pone.0234190.s008.docx]

*Dear Editor*

*We appreciate the feedback from the reviewers and the editor. We would like to take this opportunity to respond to your comments. Our responses are in italics and any new text is in bold. The lines indicated here are referred to the version with tracked changes.*

*We would be happy to provide any further information or clarification if required.*

*Sincerely*

*Kendy*

Dear Dr Teng,

Thank you for submitting your manuscript to PLOS ONE. After careful consideration, we feel that it has merit but does not fully meet PLOS ONE’s publication criteria as it currently stands. Therefore, we invite you to submit a revised version of the manuscript that addresses the points raised during the review process.
In addition to the reviewer comments listed below, I have the following comments after my own thorough reading of the paper:

Line 38: Please avoid expressions such as "positively associated with", especially in the abstract, as it is ambiguous and may be interpreted as if saying that the association is of a desirable nature.

*Authors’ response: We have modified accordingly across the manuscript. This sentence has been changed as: Being over 11 years, receiving no dry food and receiving measured amounts of feed* ***were associated with an increased odds of underweight*** *in cats (Lines 36-37).*

Line 97: Please explain the acronym BCS the first time you use it.

*Authors’ response: This has been mentioned in Line 62.*

Line 118: If the adverts did not mention obesity or overweight, how did they mention the aim of the study?

*Authors’ response: The aim mentioned on the ads is: The study is to investigate the factors that may influence the body composition of cats and people’s attitudes to the compositions.*

Line 243: "weighted" should be "weighed" (assuming that it refers to measuring the weight)

*Authors’ response: This has been modified accordingly (Line 244).*

Line 327: What does "included an increase in age before 11 years" mean? Consider rephrasing.

*Authors’ response: This has been changed to “being middle-aged” (Line 329).*

Lines 589-590: Can you please provide an example of, or consider rephrasing, "logistic regression cannot account for bidirectional causality between explanatory and outcome variables", as this statement is difficult to understand for readers who don't have a special interest in statistical methods.

*Authors’ response: We have rephrased this sentence and added an example as follows:* ***Lastly, the position of variables in logistic regression cannot imply the direction of causality. For example, in our results, frequent feeding is likely to be an owner's response to their cat’s underweight instead of the cause of underweight (Lines 592-595).***

Reviewer #1: Nice work. The manuscript is much improved, with all comments answered. Happy to accept it for publication now.

*Authors’ response: Thank you.*

Reviewer #2: The manuscript “Positive attitudes towards feline obesity are strongly associated with ownership of obese cats” by Teng et al. is an investigation on risk factors for overweight and obesity in Australian cats.

The aims of the study were to investigate:
a) “Cat owner´s attitudes towards feline overweight and obesity (O&O) and their associations with O&O in their cats
b) The risk factors for feline O&O and underweight, particularly those involving owner practice

The investigation was based on an online survey targeting the Australian cat owning population and 1,390 responses were evaluated to be valid for inclusion in the statistical analyses.

Feline obesity is a major worldwide problem and unfortunately it seems to be increasing. Therefore, it is very relevant to gain information on risk factors – to better enable successful preventative strategies. There are currently several studies investigating managerial risk factors, many of these identifying neutering and indoor confinement as major contributors to feline obesity while also differences in feeding management has been identified in several studies but results are not always agreeing. Fewer studies have focused on the owners attitudes to cats, and how owners perceive feline obesity. This is relevant information as most cats rely on their owners for food and thus owners should in most cases be able to prevent development of obesity. This study identifies attitudes that the veterinary community has to address in order to combat overweight and obesity in cats.

The manuscript is interesting and generally well written and the author has addressed most of the reviewer comments satisfactorily, improving the manuscript.

There are however, a few minor comments that could improve the clarity further, these comments refer to the manuscript version with track changes:

Abstract Line 36 and manuscript line 394: would it not be more correct to classify this risk factor as middle age as you do in the discussion ?

*Authors’ response: We have now modified accordingly (Lines 35 and 329) .*

Line 83: for owner assessed feline O&O

*Authors’ response: We have modified accordingly (Line 79).*

Line 157: their cats owner estimated body condition

*Authors’ response: We have modified accordingly (Line 151).*

Line 164-165: cats owner assessed body condition

*Authors’ response: We have modified accordingly (Line 155).*

Line 282: I am not sure the correction to weighted is right

*Authors’ response: We have changed them back to “weighed” (Line 244).*

Line 394: Fed twice daily

*Authors’ response: We have modified accordingly (Line 330).*

Line 401-404: I am not sure I understand the meaning with this sentence

*Authors’ response: It has been deleted as it was not supposed to be there.*

Line 484: BCS Vet

*Authors’ response: We have modified accordingly (Line 406).*

Table 2: could the table legend be revised to The tabulated body condition score (BCS) evaluation by the cats veterinarian and weighted kappa between different owner reported BCS estimations based on data collected by an Australian-based online performed in 2016 (n=XXX)

*Authors’ response: We have now added the number of participants in the legend. We did not include “evaluation by the cats veterinarian” as it was also owner-reported. The title is now:* ***The tabulated body condition score (BCS) evaluations and weighted kappa between different owner-reported BCS based on data collected by an Australian-based online survey in 2016 (n=1,390).***

Table 3, 4, 5 & 6: Please make sure to include in the table and figure legends both the wording Owner assessed body condition an well as the number of cats included,

*Authors’ response: We have followed the instruction for the title of tables where the information of owner-assessed BCS was included.*
